# Evaluating Introgression Sorghum Germplasm Selected at the Population Level While Exploring Genomic Resources as a Screening Method

**DOI:** 10.3390/plants12030444

**Published:** 2023-01-18

**Authors:** Noah D. Winans, Robert R. Klein, Jales Mendes Oliveira Fonseca, Patricia E. Klein, William L. Rooney

**Affiliations:** 1 Department of Soil and Crop Sciences, Texas A&M University, College Station, TX 77843, USA; 2 USDA-ARS Southern Plains Agricultural Research Center, College Station, TX 77845, USA; 3 Department of Horticultural Sciences, Texas A&M University, College Station, TX 77843, USA

**Keywords:** sorghum, introgression, genetic diversity, genomic selection, genomic resource, BC-NAM populations, hybrid performance

## Abstract

To exploit the novel genetic diversity residing in tropical sorghum germplasm, an expansive backcross nested-association mapping (BC-NAM) resource was developed in which novel genetic diversity was introgressed into elite inbreds. A major limitation of exploiting this type of genetic resource in hybrid improvement programs is the required evaluation in hybrid combination of the vast number of BC-NAM populations and lines. To address this, the utility of genomic information was evaluated to predict the hybrid performance of BC-NAM populations. Two agronomically elite BC-NAM populations were chosen for evaluation in which elite inbred RTx436 was the recurrent parent. Each BC_1_F_3_ line was evaluated in hybrid combination with an elite tester in two locations with phenotypes of grain yield, plant height, and days to anthesis collected on all test cross hybrids. Lines from both populations were found to outperform their recurrent parent. Efforts to utilize genetic distance based on genotyping-by-sequence (GBS) as a predictive tool for hybrid performance was ineffective. However, utilizing genomic prediction models using additive and dominance GBLUP kernels to screen germplasm appeared to be an effective method to eliminate inferior-performing lines that will not be useful in a hybrid breeding program.

## 1. Introduction

Grain sorghum (*Sorghum bicolor* (L.) Moench) is an important crop originally domesticated in Africa and grown in many tropical, subtropical, and temperate environments worldwide. Depending on the agricultural system, both inbred and hybrid cultivars are used with inbred varieties common in lower yield production systems and hybrids in higher yielding production systems. Grown primarily as a feed grain in the United States, sorghum covered approximately 5.8 million acres in 2020 with the top two producing states being Kansas and Texas. As with any crop, continual performance improvement is necessary to meet global demands for food and animal feed consumption and requires genetic diversity to do so [1].

Approximately 75% of the sorghum germplasm in world collections is photoperiod sensitive (PS) and unadapted to temperate-zone production. Sorghum manifests short day photoperiod sensitivity meaning that reproductive growth only initiates when daylength drops below a genetically defined level [2]. As such, PS sorghum does not initiate reproductive growth until a predefined time in the fall. In temperate production environments (i.e., greater latitudes), cool temperatures will typically inhibit growth or kill the plants prior to seed or grain production. Consequently, these PS sorghums are not adapted for use in temperate environments in the United States (US) [3]. 

In the early 20th century, sorghum improvement in the US focused on inbred cultivar development using a relatively narrow germplasm base that had been primarily selected for adaptation (i.e., earlier flowering). Once hybrid sorghum was developed [4,5], a rapidly developing sorghum breeding industry recognized the limited genetic diversity in North American germplasm. Thus, [6] hypothesized that US germplasm would benefit from diverse, tropical sorghum genes for traits such as yield and disease resistance, and to deliver these traits, they pioneered the sorghum conversion (SC) program. 

The SC program introgressed genes conferring photoperiod insensitivity (Ma1) and reduced height (Dw2) into tropically adapted germplasm to develop short, early flowering accessions suitable for temperate sorghum breeding [7]. Active from 1960 to 2003, the SC program developed and released over 700 converted lines [8,9,10]. A modified program, designated as the reinstated sorghum conversion (RSC) program, ran from 2009 to 2016 and produced another 100 lines [11]. The RSC program converted tropical accessions using molecular markers to expedite the conversion process [12]. 

Germplasm from the SC and RSC programs have increased genetic diversity in elite sorghum breeding programs while concurrently enhancing productivity of the crop. In addition to improved grain and forage quality, yield, and adaptation, the SC germplasm from these programs has provided resistance to pests, pathogens, and abiotic stresses. These lines have been used as parents in sorghum breeding programs throughout the world. From a breeding perspective, it is difficult to find a commercial sorghum hybrid produced in the past forty years that does not contain SC germplasm in its pedigree. Further, they have formed the basis for numerous sorghum genome wide association studies and have been included in many different recombinant inbred line populations for genetic mapping purposes [13,14,15,16].

While the impact of the SC and RSC programs is undeniable, some details on what germplasm was used and how it was used should influence current and future approaches to introducing and using diversity in a breeding program. For instance, over 400 germplasm and parental lines have been released from Texas A&M AgriLife Research sorghum breeding programs since 1974 [17,18,19,20]. In reviewing the pedigrees of this material, essentially all of them have at least one SC line in the pedigree. However, in all these lines, only ~30 of the over 700 converted lines appear in these pedigrees. Obviously, these 30 lines possessed desirable traits, but they also were agronomically competitive independent of any specific trait.

In addition, most breeders rarely used the fully converted germplasm, i.e., material that had gone through four cycles of backcrossing and selection in the conversion program [6]. Instead, partially converted lines (earlier backcross generation) were more commonly used in sorghum improvement programs. These partially converted lines contained a greater proportion of temperate germplasm in their pedigree, and therefore showed greater adaptation to temperate climates. This implies that the goal in using these SC lines was to introduce traits or linkage blocks that were desirable and not the recovery of the entire tropical genome per se.

A common approach to exploit the genetic diversity in unadapted germplasm while concomitantly creating a genetic mapping resource is the creation of backcross nested-association mapping (BC-NAM) populations [21,22,23]. The University of Queensland sorghum improvement program first implemented this BC-NAM approach to exploit unadapted and wild sorghum accessions for hybrid grain sorghum improvement [21]. More recently, the Texas A&M AgriLife Research sorghum improvement program implemented a similar BC-NAM approach termed the Germplasm Utilization and Enhancement of Sorghums (GUESS) program in which diversity was introgressed into both an elite B and an elite R line (based on the fertility reaction score of the unadapted parent). These sorghum BC-NAM resources represent both genetically diverse lines for sorghum improvement and a community mapping resource for geneticists who strive to elucidate those genes controlling traits of agronomic interest. 

The sorghum BC-NAM resources represent a wealth of genetic diversity, but for a hybrid grain crop such as sorghum, the value of this diversity must be evaluated in hybrid test crosses. While the evaluation of the BC-NAM populations begins with screening for agronomic fitness of the BC-NAM lines, the performance of inbred lines in most cereals is not an accurate predictor of derived hybrid performance [1]. Thus, the evaluation of genetic diversity residing in these resources must culminate in the extensive hybrid test crosses for large numbers of BC-NAM lines. This creates a daunting task for a breeding program as the number of introgression populations being created is extensive. This highlights the importance of exploring genomic resources to allow a breeding program to prescreen this germplasm before it enters the breeding program for evaluation.

The first objective of this study is to determine if there are derived lines from the populations created in the current GUESS program that can contribute to genetic gain in a sorghum breeding program. The second objective is to determine if genomic data can be used to allow a breeding program to screen through the large number of populations generated in the introgression program.

## 2. Results

### 2.1. Statistical Analysis

In the random models, pedigree was significant for all traits in both environments (Table 1). Based on the Shapiro–Wilk and Bartlett’s test, combining environments would violate the homoscedasticity of variances and normality of the residuals in subsequent analysis, leading to biased inferences (Table 1). As such, the two environments were kept separate and not combined. The Bushland environment had more genetic variation for yield than the College Station environment resulting in a higher repeatability estimate despite having a larger CVe. Alternatively, College Station had more genetic variation for plant height, which also resulted in a higher repeatability for that trait. Percent genetic variation, CVe, and repeatability were essentially equal for days to anthesis for both environments (Table 1). 

**Figure 1:**
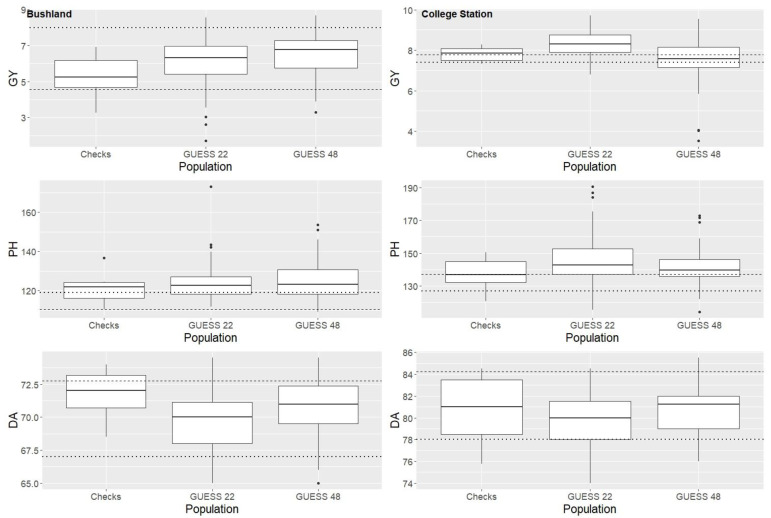
Boxplots of BLUEs for grain yield in tons/ha (GY), plant height in cm (PH), and days to anthesis (DA) in College Station and Bushland, TX. Each plot contains the distribution of BLUEs for introgression populations GUESS 22 and GUESS 48 in hybrid combination, as well as elite check hybrids from the Texas A&M AgriLife sorghum breeding program. The dashed line represents the trait mean of the recurrent parent for the introgression populations in hybrid combination, and the dotted line represents the commercial check hybrid DK37-07.

Although a small number of hybrids in the Bushland environment outperformed the commercial check DK37-07, most hybrids yielded higher than the recurrent parent in hybrid combination (henceforth, recurrent parent will refer to the recurrent parent in the experimental populations, RTx436, in hybrid combinations). In the College Station environment, the majority of GUESS 22 produced hybrids with higher grain yields compared to both the recurrent parent and the commercial check DK37-07 (Figure 1). For days to anthesis, experimental hybrid means were similar or earlier than the checks (Figure 1). Across both environments, only a few experimental hybrids flowered earlier than the commercial check, while a large percentage of experimental hybrids from both populations flowered earlier than the recurrent parent (RTx436) in hybrid combination. For plant height, the distribution of BLUEs reveals that the experimental population means were comparable to the checks, but most of the hybrids in the experimental populations were taller than their recurrent parent (Figure 1).

### 2.2. Population Structure and Genetic Distance

**Figure 2:**
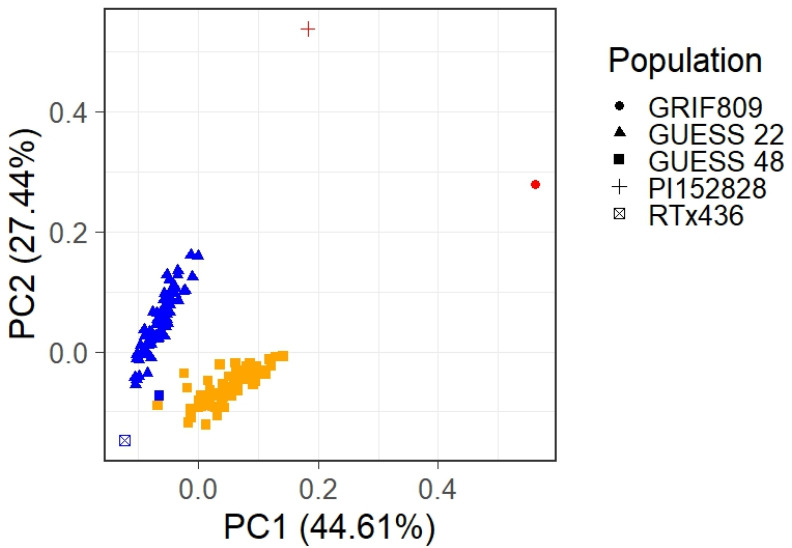
Principal coordinate analysis visualizing the relationship between the introgression populations and their respective parents. RTx436 is the recurrent parent for both populations, and PI152828 and GRIF809 are the introgression parents for GUESS 22 and GUESS 48, respectively.

PCoA revealed that each introgression population clustered between the exotic and adapted parent with a trend toward the recurrent parent (RTx436) (Figure 2). Both populations are distinct from one another with varying levels of relation to the recurrent and exotic parent within the populations. PCoA depicted the effectiveness of one backcross generation in recovering the elite (RTx436) genome while also showing that BC_1_F_3_ lines from both populations harbored novel genetic diversity introgressed from the unadapted parent.

**Figure 3:**
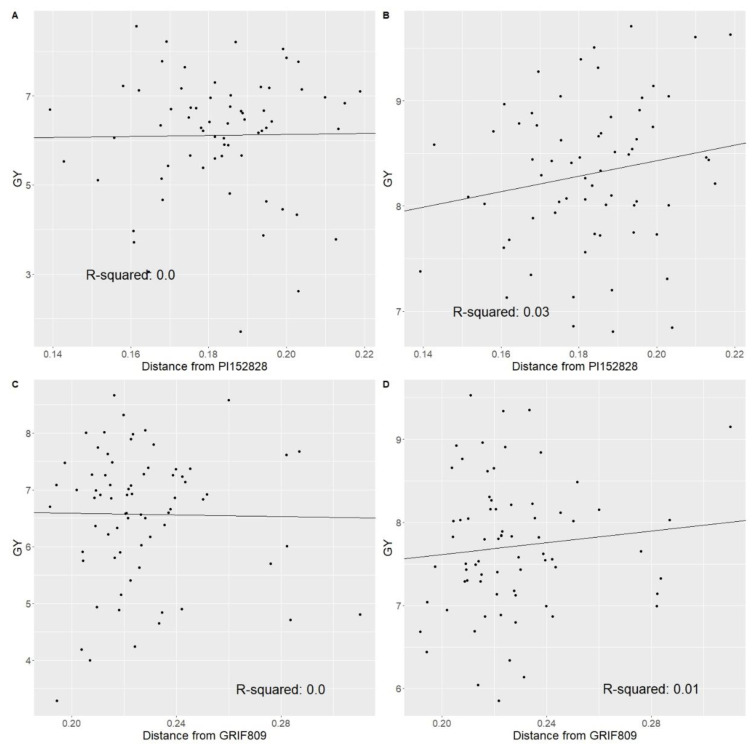
Scatterplots for determining the relationship between genetic distance to introgression parent and grain yield. (**A**) Grain yield in tons/ha (GY) BLUEs in Bushland plotted against genetic distance of GUESS 22 from PI152828 with the line of best fit. (**B**) GY BLUEs in College Station plotted against genetic distance of GUESS 22 from PI152828 with the line of best fit. (**C**) GY blues in Bushland plotted against genetic distance of GUESS 48 from GRIF809 with the calculated line of best fit. (**D**) GY BLUEs in College Station plotted against genetic distance of GUESS 48 from GRIF809 with the calculated line of best fit.

As an initial attempt to utilize genomic data to predict hybrid grain yield, the genetic distance of the BC_1_F_3_ lines from their respective unadapted parent was regressed against grain yield of their testcross hybrids. While not unexpected, genetic distance of the introgression lines from the unadapted parent was not predictive of hybrid testcross yields suggesting that the extent to which one backcross generation recovered the RTx436 genome was not predictive of testcross hybrid performance (Figure 3).

### 2.3. Genomic Prediction

#### 2.3.1. Variance Components

**Figure 4:**
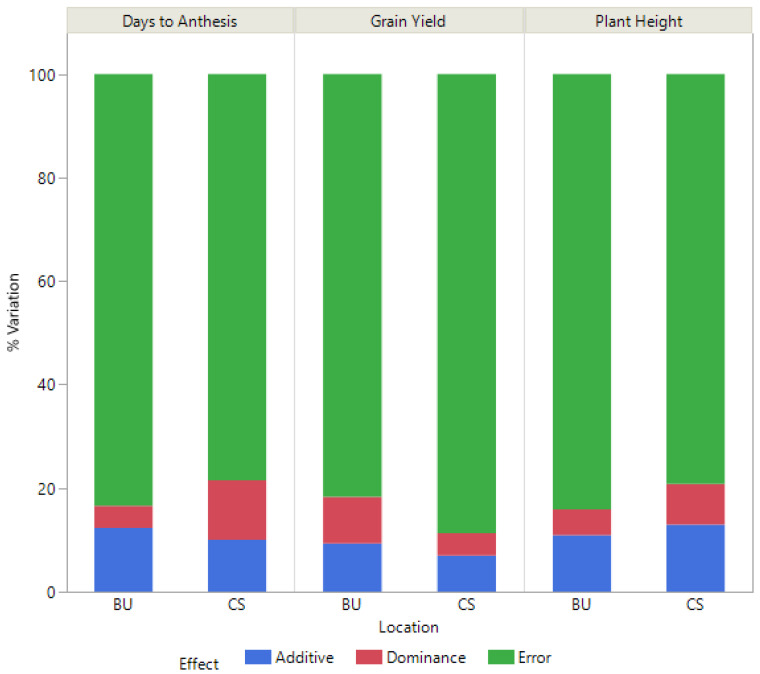
Variance components from genomic prediction models for grain yield, days to anthesis, and plant height. Percent of total variation explained (%Variation) is shown for Bushland (BU) and College Station (CS) environments with variation partitioned to additive, dominance, and residual effects (Error).

To understand how the genomic prediction models are explaining variation, phenotypic variation explained by the models was partitioned into additive, dominance, and residual effects. In both environments, more of the genetic variation for yield was partitioned to additive than dominance effects (Figure 4). For days to anthesis, more of the variation was attributed to genetic effects in College Station than in Bushland and was equally split between additive and dominance effects in College Station while being partitioned more to additive effects in Bushland (Figure 4). Plant height was like grain yield with additive effects explaining more of the variation than dominance and more variation was attributed to genetic effects in College Station than Bushland (Figure 4).

#### 2.3.2. Cross-Validation Scheme CV1

The results using cross-validation scheme CV1 (50% of the introgression lines were randomly selected to be the training set and the remaining 50% were the validation set) are shown in Table 2. For grain yield, higher correlations between predicted and observed data were seen in Bushland than in the College Station environment. This resulted in a concurrent increase in the accuracy of predicting the top performing 15% of the lines in Bushland relative to College Station. In contrast, there was no improvement in the prediction accuracy of eliminating the bottom performing 50% of the lines in Bushland over College Station. Alternatively, correlations between predicted and observed plant height and days to anthesis were higher in College Station than in Bushland. Prediction accuracy for plant height and days to anthesis were greater than prediction accuracy of grain yield in College Station; however, this was not the case in Bushland (Table 2).

#### 2.3.3. Cross-Validation Scheme CV3

**Figure 5:**
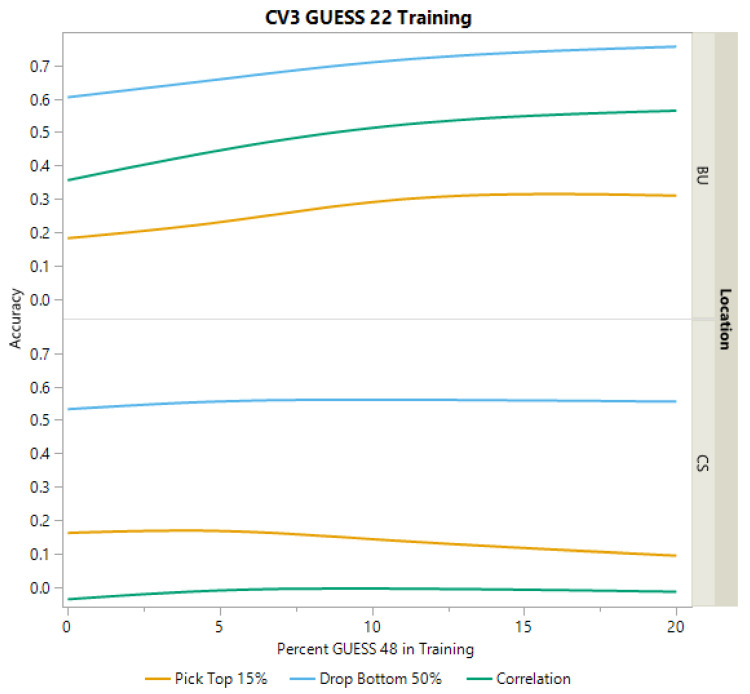
Prediction accuracies for CV3 in Bushland (BU) and College Station (CS). Accuracies for grain yield are presented with varying levels of GUESS 48 being added to the training data that include all of GUESS 22. Accuracy for grain yield was assessed in three ways, the proportion of hybrids correctly predicted in the top 15%, the proportion of hybrids correctly predicted in the bottom 50%, and correlation between predicted and actual values.

**Figure 6:**
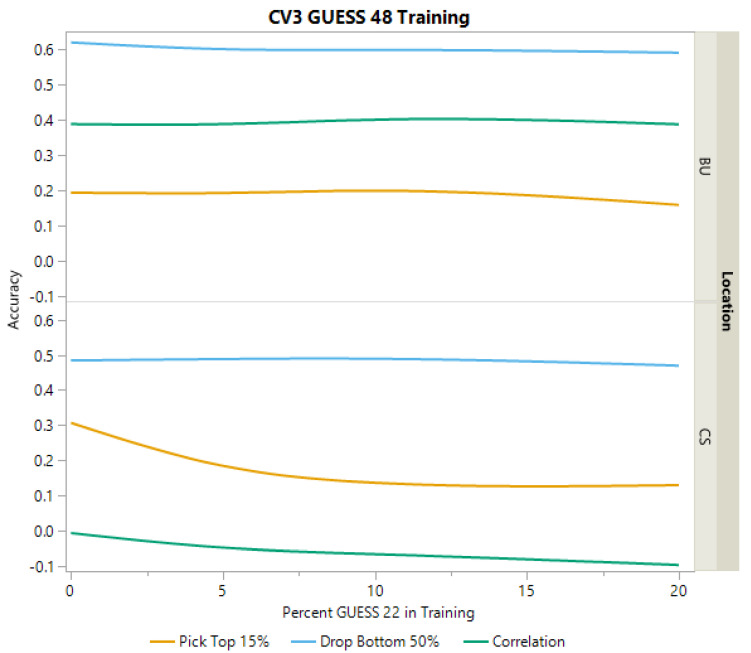
Prediction accuracies for CV3 in Bushland (BU) and College Station (CS). Accuracies for grain yield are presented with varying levels of GUESS 22 being added to the training data that include all of GUESS 48. Accuracy for grain yield was assessed in three ways, the proportion of hybrids correctly predicted in the top 15%, the proportion of hybrids correctly predicted in the bottom 50%, and correlation between predicted and actual values.

As both BC-NAM populations utilized a common recurrent parent (RTx436), cross-validation scheme (CV3) was implemented to attempt to predict performance of a population using a subset of lines from the population of interest along with the complete dataset of the second population as training data. Despite sharing a common recurrent parent, cross-validation scheme CV3 showed poor predictive performance for grain yield accuracies in the College Station environment. For both populations, correlations were near zero and the ability to select the top 15% remained low as well as the ability to eliminate the bottom 50% (Figure 5 and Figure 6). In the Bushland environment, CV3 was more effective in predicting the performance across populations (Figure 5). For example, when using GUESS 22 to predict GUESS 48, as a greater proportion of GUESS 48 was added to the training data, the ability to identify the bottom 50% and correlations are similar or slightly better than those observed in CV1. However, the ability to pick the top 15% using cross-validation scheme CV3, remained lower than that observed for CV1 (Table 2, Figure 5). While improvements in prediction can be seen as more of GUESS 48 is added to the training set, it does appear that the increases plateaued between 10% and 20% (Figure 5). When using GUESS 48 to predict GUESS 22 in Bushland, correlations and the ability to drop the bottom 50% remained like accuracies observed in CV1; however, no increase was seen from adding more of GUESS 22 to the training data (Figure 6).

## 3. Discussion

The purpose of BC-NAM populations is to introgress genetic diversity into elite lines. The unadapted lines chosen for the GUESS program harbor desirable traits (i.e., panicle architecture, abiotic/biotic stress tolerance) believed to be of value to improve elite sorghum germplasm. The populations underwent strong selection pressure for agronomic fitness (i.e., maturity, height, standability) by a panel of plant breeders. Due to this, the populations created in the GUESS program all exhibit potential and would be deemed acceptable to use in a prebreeding program such as the two observed herein. Despite these efforts, the value of an inbred sorghum is determined by its hybrid performance in combination with elite testers. This results in a daunting task for a breeding program due to the ~30 BC-NAM populations perceived to have breeding value that have been generated in the GUESS program. Thus, exploring the possibility of utilizing genomic resources for this germplasm is important for beginning to sort through the large numbers of introgression lines that have been generated in the GUESS program.

### 3.1. BC-NAM Genetic Diversity and Hybrid Performance

While PCoA was useful for visualizing the relationships between the derived populations and their respective parents, genetic distance was not an effective predictor of hybrid performance. A study by [24] found a significant effect between yield and heterozygosity while assessing genetic distance using restriction fragment length polymorphism markers, yet such significance did not identify high-yielding hybrids. More recently, [1,25] reported that genetic distance of inbred parents is not an effective predictor of hybrid performance. While the methodology differs, the results in this study support that genetic distance, albeit in the distance of derived lines from their recurrent parent, is a poor predictor of hybrid performance. While it is a poor predictor of hybrid performance, PCoA and genetic distance can help visualize the genetic diversity among novel genetic mapping populations in relation to lines presently utilized in a breeding program. Additionally, PCoA can assist breeders in making more informed decisions in breeding crosses within heterotic groups [26] and aid in selecting training data for genomic prediction [27].

The range of BLUEs for grain yield in both environments revealed that developing BC-NAM populations using an elite recurrent parent can produce lines that outperform commercial inbreeds in hybrid combination while also incorporating novel diversity to the breeding program. Additionally, the distributions of BLUEs for plant height and days to anthesis demonstrated that lines from these populations produce agronomically acceptable hybrid combinations. A study by [21] reported similar results in BC-NAM populations developed by the University of Queensland sorghum improvement program. While elite material is present in these sorghum BC-NAM populations, their frequency demands the development of alternative approaches to clearly delimit these superior lines while minimizing field testing of all the potential progeny derived from these introgression populations.

The populations evaluated herein were R-lines based on fertility restoration and genetic distance, which in turn will result in an increase in the favorable alleles within the R line heterotic pool. The genetic relatedness of the unadapted, nonrecurrent parent was compared to a panel of elite R and B lines to ensure that alleles from the nonrecurrent parent would not compromise the genetic distinctness of sorghum heterotic pools (B and R-lines) [28]. Herein, only R-lines were considered; if a similar study was performed on the second heterotic pool (B lines) of sorghum the introgression of unique and favorable alleles could increase heterosis due to the complementary action of dominance effects. 

### 3.2. BC-NAM Populations and Genomic Prediction

Using genomic prediction as a screening method for introgression material would allow breeding programs to evaluate and prescreen diverse material to integrate into the breeding pipeline. Results herein indicated that the two environments examined (College Station, Bushland) were very distinct and as such, it was more effective to make predictions in an environment-specific approach. 

Although genomic prediction models produced modest correlations, genomic selection could still be effective for eliminating those parental inbred that generated the poorest performing hybrids. Models with modest correlations were not as applicable to identify the top performing hybrids because, as correlations drop, there is a sharp decline in the percentage of hybrids correctly predicted to be in the top 15%. However, the prediction accuracy of the poorest performing hybrids (i.e., >50%) was consistent. Thus, genomic prediction may be an effective strategy to reduce the amount of field evaluation by identifying and eliminating, in silico, hybrids performing below average. The present results were consistent with [29] who proposed that breeders are unlikely to use only genomic selection for selecting the best hybrids, but are more likely to use genomic prediction as a means of eliminating the least promising material from field evaluation. The results presented herein support this proposal by demonstrating the difficulty in predicting the highest performing individuals in a population, but the apparent ability to predict the bottom half of a population with acceptable accuracy.

An alternative approach to intrapopulation (CV1) predictions is the use of interpopulation predictions (CV3) wherein a base population (GUESS 22 or GUESS 48) combined with (or without) a subset of each of the other populations would also be effective to eliminate the bottom 50% of individuals from the remaining populations. This would greatly reduce the number of individuals that need to be testcrossed across populations and would provide an effective way for breeding programs to begin evaluating large numbers of introgression populations and lines in silico. Previous studies have reported negligible genomic prediction accuracies when heterogeneous populations are utilized for genomic predictions studies. A study by [30] found in rice and maize that genomic prediction across diverse panels resulted in poor prediction accuracy even with corrections for population structure. 

In the current study, the backcross-derived populations created with a common elite recurrent parent have a greater genetic relatedness compared with NAM (or unrelated RIL populations) and thus, offer a greater opportunity to utilize a joint genomic prediction approach (i.e., prediction using multiple introgression populations) to increase the power of prediction. A joint genome prediction approach represented in cross-validation scheme CV3 would partially alleviate the limitation imposed by the relatively small population sizes (e.g., ≤100 lines per population) normally associated with nested-association mapping populations where the number of populations is often maximized and the number of lines per population reduced. The present joint genomic prediction effort demonstrated that it is possible to predict across diverse backcross-derived populations in certain situations especially if some genotypes from the target population are present in the training set. 

For the cross-validation scheme CV3 in Bushland, a training set consisting of GUESS 22, combined with a small subset of GUESS 48, increased the prediction accuracy to identify in silico the bottom 50% when compared to cross-validation scheme CV1. However, this was not consistent across environments as the same improvement in prediction accuracy with CV3 vs. CV1 was not seen in College Station. Furthermore, it should be noted that there is a population-effect contributing to prediction accuracy as an increase was not observed in both cross-validation scenarios of CV3 in Bushland. Future efforts are required to understand those factors contributing to this population effect and thereby allow for more effective implementation of genomic prediction of diverse populations in sorghum hybrid improvement programs. Finally, the importance of highly repeatable data such as those observed in Bushland was evident in the ability to predict hybrid performance across populations while the poorer prediction accuracies observed in College Station are consistent with other studies.

## 4. Materials and Methods

### 4.1. Population Development

The genetic material for this study consists of two BC_1_-NAM populations from the Texas A&M AgriLife Research GUESS program test-crossed to elite female (male-sterile) lines. The two populations were selected from a total of 30 BC_1_-NAM populations based on their agronomic fitness and breeding desirability (as determined by Texas A&M AgriLife Research Sorghum Improvement) [28]. As both populations are derived from unadapted R lines backcrossed to RTx436, each BC_1_F_3_ line within both populations was test-crossed to an elite A-line to produce the experimental hybrids.

The first population (GUESS 22) consists of 68 BC_1_F_3_ lines with the pedigree of RTx436ms3//RTx436ms3/PI152828. The second population (GUESS 48) consists of 70 BC_1_F_3_ lines with the pedigree of RTx436ms3//RTx436ms3/GRIF809. Throughout population development, lines were selected for agronomic quality based on traits such as plant height, absence of tannins, panicle exsertion, and days to flower. Lines from both populations were hybridized to either ATx2928 or A03017 (elite female from the program) for a total of 143 experimental hybrids with 75 from GUESS 48 (5 lines were hybridized to both A-lines) and 68 from GUESS 22. Check hybrids included were ATx2928/RTx2783, ATx2928/RTx436, and ATx2928/RTx437 and A03017/RTx2783, A03017/RTx436, and A03017/RTx437.

### 4.2. Hybrid Evaluation

The hybrids were grown in two distinct environments over two years. In each location, entries within a population were arranged in randomized complete block design with two replicates. The 2020 environment was College Station, TX (30.5488, −96.4361), and the 2021 environment was Bushland, TX (35.2146, −102.0627). Locations were managed with agronomic practices representative of the area, with the College Station environment being rainfed and the Bushland environment being on limited irrigation. 

Three agronomic traits were evaluated in each environment. Days to anthesis (DA) were recorded as the number of days for 50% of the plants to reach mid-anthesis. Terminal plant height (PH), measured from the soil at the base of the plant to the top of the panicle, was collected before harvest using a representative plant from the plot. Grain yield (GY), plots were harvested using plot combines and GY (tons∙ha^−1^) adjusted to a 14% moisture content.

### 4.3. Genotype Data

DNA from the parental accessions, elite inbred checks, and BC_1_F_3_ NAM lines was extracted from leaf tissue of seedlings. Briefly, for the high-throughput genotyping-by-sequencing (GBS) method, genomic DNA from each line was extracted and digested with a methylation-sensitive enzyme *Ngo*MIV, and an Illumina template library was prepared as described by [31] with single-end sequencing performed on the Illumina NovaSeq 6000 (Texas A&M AgriLife Genomic and Bioinformatics Services). The processing of sequencing data and variant detection based on mapping to the reference BTx623 genome (Sbicolor 3.1) was as described by [32]. After imputation, markers with marker allele frequency of less than 5% were removed leaving 67,315 SNPs for further genetic analysis.

### 4.4. Statistical Analysis

Statistical analysis were carried out in R [33] using the *lme4* package [34] to fit a random coefficients model for each environment as follows:

(1) $$y=1\mu +Z_{1}h+Z_{2}r+\varepsilon$$

where y represents the vector of phenotypic records for the trait of interest (*n* = 279 for BU, *n* = 314 for CS); μ represents the mean; h represents the effect of hybrids, $h\;\sim \left(0,\sigma_{h}^{2}\right)$; r represents the effect of replicate, $r\;\sim \left(0,\sigma_{r}^{2}\right)$; ε represents residual error, $\varepsilon \;\sim \left(0,\sigma_{\varepsilon }^{2}\right)$; 1 represents a vector of ones; $Z_{1}$ and $Z_{2}$ represent incidence matrixes; $\sigma_{h}^{2}$, $\sigma_{r}^{2}$, and $\sigma_{\varepsilon }^{2}$ represent variance components for hybrids, replicates and residuals, respectively. The significance of effects were assessed using Wald’s *p*-value [35], and percent of variation explained by the hybrids were presented. A similar model assuming the effect of hybrid as fixed was also used for extracting Best Linear Unbiased Estimates (BLUEs) to compare agronomic performance of GUESS 22, GUESS 48, and checks in hybrid combination. Calculated BLUEs were used to develop genomic prediction models.

To assess the possibility of combining environments, the Shapiro–Wilk test [36] was used to evaluate the normality of residuals, and Bartlett’s test [37] was used to assess the homogeneity of residual variances. 

### 4.5. Population Structure Analysis

Principal coordinate analysis (PCoA) was used to visualize the relatedness of the populations to each other, as well as to their unadapted and elite parents as described by [26]. Genetic distance of the lines in each population to their respective introgression parent was calculated using TASSEL5 [38]. The correlation from a linear regression was then used to determine if there is a relationship between the genetic distance of the lines from their introgression parent with performance in the form of hybrid grain yield.

### 4.6. Genomic Prediction

Genomic prediction was done using a hierarchical Bayesian framework in the R package *BGLR* [39] using GBLUP kernels. The covariance matrix for GBLUP was calculated as [40]:

(2) $$K_{G}=\frac{XX^{\prime }}{2\sum p_{i}\left(1-p_{i}\right)}$$

where *K_G_* is the Genomic Relationship Matrix (GRM), *X* is the marker matrix of the form n (individuals) x p (markers), and *p_i_* is the frequency of the second allele at locus *i*. This describes the calculation of covariance matrices based on additive effects for the parental lines. Dominance effects were also calculated following [41] using the *AGHmatrix* package in R [42]. These were then used to create the GRM for the hybrids for both additive and dominance effects by calculating the Kronecker product of the parental covariance matrices.

For individual environment predictions, the model was of the form:

(3) $$y=\mu +Z_{A}u_{A}+Z_{D}u_{D}+\varepsilon$$

where y represents a vector of phenotypic values (*n* = 148), μ represents the mean, $u_{A}$ represents additive effects, $u_{A}\sim N\left(0,K_{A}\sigma_{A}^{2}\right)$ where $K_{A}$ is the additive GRM, $\sigma_{A}^{2}$ represents the variance component associated with additive effects; $u_{D}$ represents dominance effects, $u_{D}\sim N\left(0,K_{D}\sigma_{D}^{2}\right)$ where $K_{D}$ is the dominance GRM, $\sigma_{D}^{2}$ represents the variance component associated with dominance effects. $Z_{A}$ and $Z_{D}$ represent the incidence matrices of additive and dominance effects, respectively, and ε represents residual error. It should be noted that $Z_{D}$ and $Z_{A}$ are identical, and ⊗ represents the calculation of the Kronecker product. 

To assess the prediction accuracy of models and simulate strategies to screen lines, two schemes were utilized. The first cross-validation scheme (CV1) predicts the value of new lines for inclusion in the breeding program. In CV1, 50% of the introgression lines were randomly selected to be the training set; the remaining 50% were used for the validation set. This was repeated 20 times and the prediction accuracy was assessed using three metrics for GY: correlation between predicted and observed values, percent accuracy in selecting the top 15% of hybrids, and percent accuracy in dropping the bottom 50% of hybrids. Prediction accuracy for PH and DA were assessed using correlation between predicted and observed values. CV1 was used for predictions in a single environment. The other cross-validation scheme (CV3) simulates using one population with a subset of the other to predict the rest of the incomplete population. CV3 was used with varying levels of 0%, 5%, 10%, and 20% training data coming from the predicted population. CV3 was used for single environment predictions with 20 repetitions using the same metrics for accuracy as in CV1.

## 5. Conclusions

Genetic mapping populations such as NAM and BC-NAM represent a rich source of genetic diversity that has been instrumental in mapping trait loci controlling various phenotypes of interest to geneticists and crop improvement programs. While these resources still represent a powerful tool for mapping trait loci, their value to most crop improvement programs has diminished due to the general lack of applicability of QTL analyses in hybrid improvement efforts. However, depending on the populations created and the choice of recurrent parent, superior lines can be identified within NAM and BC-NAM populations. By implementing a strategy of genomic prediction, it appears feasible to eliminate the poorest performing lines within these mapping resources without extensive field testing and thereby reduce the inputs required to evaluate these lines in hybrid combination. While it may be difficult to utilize genomic prediction to accurately predict the most desirable hybrid combinations, the results herein suggest that genomic prediction could be successfully employed to discard the least desirable experimental lines. Nevertheless, this strategy must still be cautiously implemented and critically evaluated due to the risk of Type II error.

## Figures and Tables

**Table 1:** Variance components, repeatability (R), and coefficient of variation (CVe) estimates derived from single environment analysis of grain yield, plant height, and days to anthesis. The percent of total variation (%) for hybrids, replicate, and residual are shown with their respective estimates. Wald *p*-value was used to indicate significance of effects. Shapiro–Wilk (W) and Bartlett test (F-test) followed by their corresponding *p*-values are presented. *, **, and *** represent significance at the 0.05, 0.01, and 0.001 level, respectively.

**Variance Component**	**College Station**					
	**Grain Yield**		**Plant Height**		**Days to Anthesis**	
	**Estimate**	**%**	**Estimate**	**%**	**Estimate**	**%**
$\sigma_{h}^{2}$	0.28 ***	27.7	142.9 ***	75.4	4.91 ***	70.1
$\sigma_{r}^{2}$	0.09	8.8	1.3	0.7	0.06	0.9
$\sigma_{\varepsilon }^{2}$	0.65	63.5	45.4	23.9	2.03	29.0
CVe	10%		4.72%		1.80%	
R	0.466		0.863		0.829	
**Variance Component**	**Bushland**					
	**Grain Yield**		**Plant Height**		**Days to Anthesis**	
	**Estimate**	**%**	**Estimate**	**%**	**Estimate**	**%**
$\sigma_{h}^{2}$	1.19 ***	54.3	41.4 ***	35.8	4.07 ***	73.41
$\sigma_{r}^{2}$	0.15	6.6	8.9	7.7	0.002	0.03
$\sigma_{\varepsilon }^{2}$	0.86	39.1	65.4	56.5	1.47	26.56
CVe	14.8%		6.45%		1.70%	
R	0.735		0.559		0.847	
**Test**	**W**		**F**		*p*-value	
Shapiro–Wilk	0.99		-		0.0021 **	
Bartlett	-		1.2466		0.021 *	

**Table 2:** Prediction accuracies for single environment under cross-validation scheme CV1 using model M1. Mean correlations followed by standard errors are presented for grain yield (GY), plant height (PH), and days to anthesis (DA). Additionally, the accuracy to predict the top performing 15% and bottom performing 50% for YD are reported.

College Station	YD Correlation	PH Correlation	DA Correlation	Top 15% YD	Bottom 50% YD
Mean	0.179	0.364	0.633	0.132	0.599
Standard Error	0.048	0.081	0.044	0.081	0.047
**Bushland**					
Mean	0.485	0.143	0.257	0.332	0.703
Standard Error	0.069	0.115	0.098	0.085	0.071

## Data Availability

Genotypic data can be found at [28] Phenotypic data can be found at https://github.com/ndwinans/Introgression_Data.
